# Implementation matters: assessing the effectiveness and sustainment of an obstetric triage program at a high-volume facility in Ghana

**DOI:** 10.1186/s43058-023-00527-y

**Published:** 2023-11-15

**Authors:** Rohit Ramaswamy, Stephanie Bogdewic, Caitllin R. Williams, Sylvia Deganus, Gifty Ama Bonzi, Joana Boakye, Emelia Koranteng, Rosemond Mensah, Alice Amanor, Fiona Bryce, Medge D. Owen

**Affiliations:** 1 James M. Anderson Center for Health Systems Excellence, Cincinnati Children’s Hospital Medical Center, Cincinnati, OH USA; 2 Department of Maternal and Child Health, Gillings School of Global Public Health, University of North Carolina at Chapel Hill, Chapel Hill, NC USA; 3 Tema General Hospital, Ghana Health Service, Tema, Ghana; 4 Kybele, Inc., Lewisville, NC USA; 5 Department of Anesthesiology, Wake Forest School of Medicine, Winston-Salem, NC USA

**Keywords:** Ghana, Low- and middle-income countries, Obstetric triage, Scale-up, Theory of change, Maternal newborn health, Implementation theory, Evaluation

## Abstract

**Background:**

Maternal mortality remains stubbornly high in Ghana. Current national efforts are focused on improving the quality of care offered in health facilities. Obstetric triage is one intervention that has been proposed to improve the timeliness and appropriateness of care, two key elements of quality. In this study, we describe and evaluate a theory-based implementation approach to introduce obstetric triage into Tema General Hospital, a high-volume maternity hospital in Greater Accra, that blends concepts from implementation science and quality improvement. This implementation project was a first attempt to scale this intervention into a new facility, following initial development in the Greater Accra Regional Hospital (formerly Ridge Hospital) in Accra.

**Methods:**

This was a retrospective mixed-methods evaluation of two stages of implementation: active implementation and sustainment. We triangulated monitoring data captured during active implementation with clinical outcome data (timeliness of first assessment, accuracy of diagnosis, and appropriateness of care plan) from direct observation or patient obstetric triage assessment forms at baseline, at the completion of the active implementation stage, and following a 12-month “washout” period with no contact between hospital staff and the purveyor organization. Finally, we assessed embeddedness of the new triage procedures using the NoMad, a quantitative assessment of constructs from normalization process theory (NPT).

**Results:**

Patient waiting time decreased substantially during the study. At baseline, the median arrival-to-assessment waiting time was 70.5 min (IQR: 30.0–443.0 min). Waiting time decreased to 6.0 min (IQR: 3.0–15.0 min) following active implementation and to 5.0 min (IQR: 2.0–10.0 min) during the sustainment period. Accuracy of diagnosis was high at the end of active implementation (75.7% correct) and improved during the sustainment period (to 77.9%). The appropriateness of care plans also improved during the sustainment period (from 66.0 to 78.9%). Per NoMad data, hospital staff generally perceive obstetric triage to be well integrated into the facility.

**Conclusions:**

This theory-based implementation approach proved to be successful in introducing a novel obstetric triage concept to a busy high-volume hospital, despite resource constraints and a short implementation window. Results proved long-lasting, suggesting this approach has high potential for engendering sustainability in other facilities as well. Our approach will be useful to other initiatives that aim to utilize program data to create and test implementation theories.

**Supplementary Information:**

The online version contains supplementary material available at 10.1186/s43058-023-00527-y.

Contributions to the literature

- This article responds to recent calls to bring methods of implementation science to address real world healthcare challenges. It demonstrates the use of a theory-based approach to develop site-specific implementation strategies to improve adoption of an evidence-based intervention.
- It provides an example of how implementation science and quality improvement approaches can be integrated, resulting in positive outcomes
- This article contributes to the nascent literature on defining and measuring sustainment. Sustainment was measured 1 year beyond the initial intervention with results supported by normalization process theory.
- The paper demonstrates an example of a “theorizing” approach where data from the evaluation of a prior implementation is used to create and test a higher-level implementation theory.

## Introduction

Reducing maternal and neonatal mortality remains a priority across Sub-Saharan Africa. Between 2000 and 2017, sub-Saharan Africa achieved a 40% reduction in maternal mortality [1]. Neonatal mortality declined by 54% between 1990 and 2020 [2]. Despite these impressive achievements, maternal and neonatal mortality remain elevated in sub-Saharan African countries relative to the global average. Although facility-based births have continued to increase, mortality rate reductions have stagnated, suggesting gaps in the provision of quality care. The need for innovations is especially critical in high-volume referral hospitals that care for the highest risk mothers and where mortality is higher than the national norm [3].

Innovations on their own are useless without their effective and sustained implementation. The field of implementation science provides theories, models, and frameworks to assist in the development of systematic approaches to implementation [4]. However, these theories, models, and frameworks have been difficult to utilize effectively in practice settings partly because there has been limited guidance on how to develop and measure the effectiveness of implementation processes [5, 6]. In this paper, we describe and evaluate the application of a structured approach to implement obstetric triage, an innovation developed to reduce delay and prioritize care for high-risk mothers in high-volume facilities by the Ghana Health Service (GHS) and Kybele Inc., a US- and Ghana-based non-governmental organization.

### Background and history of obstetric triage

The obstetric triage program was developed and implemented at the Greater Accra Regional Hospital (GARH) in 2013 [7, 8]. This outcome primarily focused on reducing waiting time and on testing and finalizing the clinical components of the triage system using a quality improvement (QI)-based implementation strategy. Between 2013 and 2015, the median time between arrival and assessment decreased from 40 to 5 min. [3, 7]. The GARH program, which was implemented over 4 years, also led to recognition that scaling the program to other facilities would require a more systematic approach and the development and testing of formal strategies to implement the key steps of the triage process with fidelity. In 2018, an opportunity to implement at a second high-volume facility in the Greater Accra region allowed us to do this. In this paper, we describe the implementation approach at this facility and evaluate the achievement of outcomes, the assessment of implementation quality, and the sustainment of key program indicators 12 months following the end of formal implementation support. Our results demonstrate the utility of systematic implementation science-based approaches to improve implementation quality and sustainment of global health programs. Our findings and recommendations can continue to strengthen the development and use of implementation theories to guide implementation in the field.

## Methods

### Study setting

The implementation took place at Tema General Hospital, a high-volume hospital in the Greater Accra Region of Ghana, serving a population of one million. The hospital functions as a primary care facility as well as the main referral center for surrounding areas. Maternity services comprise one of the largest and busiest departments in the hospital, including eleven sub-units. In 2017, the maternity department received nearly 1200 referrals and reported 6458 deliveries; between the years 2013 and 2017, the department averaged 6772 deliveries per year. At the time of the program implementation in 2018, the facility employed 103 nurses and midwives in maternity services.

### Intervention

The triage intervention followed the steps developed at GARH as previously reported [8]. The implementation was led by *triage champions*, who were selected based on criteria developed during the team’s experience at GARH (provided in Supplemental File 1) that included midwifery experience in the antenatal clinic and labor ward, with proven communication and leadership skills. Following an initial site visit by Kybele team members in June 2018, the facility leaders were asked to select four midwives to serve as champions. In September 2018, Kybele team members and a triage champion from GARH trained the champions over the course of 2 days through didactic lectures, small group discussions, and role play.

The training included an introduction and application of triage concepts in maternity care, information on equipping a triage unit, and instruction on the four key steps of the triage process: (a) performing a clinical evaluation of the patient and completing an assessment form; (b) categorizing the patient’s risk status as low, medium, or high risk; (c) applying a color-coded wristband to the patient corresponding with risk; and (d) creating a care plan aligned with the patient’s condition and risk level. The champions were also provided with a triage toolbox that included colored wristbands and a triage risk acuity chart, triage assessment forms, guidelines for establishing and equipping a triage space, and a triage job aid with clinical protocols for midwives [8]. After training, champions engaged in a full day of on-site mentoring to practice what they had learned in the triage area and were observed and coached by the clinical training team.

The triage champions in turn trained a group of 15 peers including ten midwives from the antenatal clinic, antenatal ward, and labor ward, as well as a recovery nurse, neonatal intensive care unit nurse, medical officer, nurse anesthetist, and the deputy director of nursing of over a period of 2 days. Kybele team members observed this training and provided support as needed. Additionally, the champions received guidance on how to maintain the triage room, how to provide on-going clinical coaching, and how to provide refresher trainings.

### Implementation approach

The inadequacy of training alone to achieve outcomes has long been recognized. Implementation researchers have identified the need for training to be reinforced with tools, ongoing support, and a process for quality assurance and improvement of implementation [9].

We used the list of common strategies compiled by the Expert Recommendations for Implementing Change (ERIC) study [10], and their stratification by Leeman et al. (2017) [11] by the actors who are responsible for them, as the basis for developing our implementation approach. Based on our experience from the GARH implementation, we developed a “capacity building” strategy enacted by the clinical champions who are implementation support system actors. From the ERIC list, the specific strategy selected was “developing and implementing tools for quality monitoring.” Although we selected a single implementation strategy to categorize our efforts at Tema General Hospital, we acknowledge the multifaceted nature of implementation strategies and that our “capacity building” strategy incorporates elements of data monitoring, coaching, and quality improvement.

This strategy was operationalized by using the Model for Improvement, which is a widely used model for healthcare quality improvement [12]. The model leads QI teams through creating a hypothesis for change, developing measures, creating change ideas, and iteratively testing them though the Plan/Do/Study/Act (PDSA) cycle. Champions were trained on using the Model for Improvement to set implementation fidelity targets, monitor progress relative to these targets, and develop and test solutions to address barriers to implementation.

### Implementation monitoring

The formal implementation support provided by the coaches was planned for 90 days after training. In this period (September 24–December 1, 2018), the implementation strategy focused on two implementation *compliance* indicators shown in Table 1, because triage could not take place without these two steps. These monitored whether the patients were banded and whether the assessment forms were completed. Detailed instructions were provided on how to collect data manually on a small sample of patients each week and how to plot the data on a run chart to measure the performance trend over time (this is a standard QI approach) [13]. To monitor compliance to banding, data was collected at the beginning of each shift, with the champions counting the number of banded patients in ward. Results where aggregated weekly for reporting. To monitor whether the triage form was being completed, 10 assessment forms were randomly selected every week and audited. To reduce the burden on the midwives, champions collected the data and sent it by WhatsApp to a member of the Kybele implementation team who updated and maintained the plots. Champions monitored weekly performance of these indicators on the run charts and used PDSA thinking to plan system improvements to get them as close to perfection as possible.

**Table 1:** Implementation compliance indicator summary

Stage	Definition	Data collection and sample size	Indicator	Duration
Banding compliance	The extent to which patients in the ward, during a given shift, are currently wearing a colored wristband to demonstrate risk acuity (high risk, intermediate risk, or low risk)	Manual count of patients in the ward at the beginning of each shift	% of clients in ward wearing a colored wristband, averaged across all shifts in a week	Weekly until 90 days after training completion
Triage assessment form compliance	The extent to which the triage assessment form is completed, that is, no missing or incomplete fields	Random sample of 10 triage forms from the preceding week	Average % of triage assessment form fields completed in the sample	Weekly until 90 days after training completion

After the 90 days, champions were encouraged to continue monitoring these indicators for another 6 to 8 weeks and to coach staff to improve performance on these indicators. Formal use of PDSA cycles was not mandated for this period. In addition, the champions were encouraged to pay attention to two indicators for implementation *quality*: (a) the percent of patients who were banded correctly based on their clinical assessment at triage and (b) the percent of patients whose care plan was appropriate to their risk assessment because these would be part of the triage evaluation. Since the monitoring requirements for these indicators were more labor intensive (requiring a clinical review), they were not mandated. Nevertheless, some champions collected and reported the data and run charts for all four indicators were created until early January 2019.

### Evaluation

The evaluation of the triage implementation was conducted using data collected at baseline (prior to implementation), immediately following implementation, and 12 months after. The following data was collected to measure outcomes, implementation quality, and sustainment.

#### Outcome data

As mentioned previously, the primary outcome was the elapsed time from the arrival of the patient into the facility to when the patient was assessed and given a wristband. The U.S. Association of Women’s Health, Obstetric and Neonatal Nurses recommends that triage be initiated within 10 min of a woman’s arrival to a facility [14]. Consequently, the indicator for this outcome was defined as the percent of women assessed within 10 min of arrival. We also examined overall waiting time. Data for this indicator was collected at three points during the project: at baseline prior to the implementation, after implementation was complete, and 12 months following the end of implementation. Since the triage program had not been established, a pragmatic approach was needed for baseline data collection. A midwife at the hospital recorded when the patient arrived and when she was first seen by a midwife for approximately two weeks before the training began.

Our data collection strategy was based on practical considerations related to resource availability. At baseline, data collection involved observation and manual recording of arrival and assessment time by a staff midwife. Post-implementation, it involved acquiring a random sample of patient files and extracting data from them. Data was collected on 66 women at baseline. After the training, the arrival and assessment times were obtained from the triage assessment form. A random sample of 103 forms was collected after implementation (November 2018–January 2019; time data was incomplete on one form). A random sample of 104 triage forms from December 2019 to February 2020 was used for the sustainment period. At 95% confidence and 80% power, the sample size of 66 observations at baseline would be more than adequate to detect a post-implementation increase similar to that in GARH (65% seen within 10 min up from a baseline of 22%) [7]. Since the same intervention was implemented in this study, there is no reason to expect a different result.

#### Implementation quality data

There were two indicators for implementation quality: (a) the percent of patients who were banded correctly based on their clinical assessment at triage and (b) the percent of patients whose care plan was appropriate to their risk assessment. The assigned band and the care plan established for the patient were also routine entries in the triage assessment forms. The 103 forms collected between November 2018 and January 2019 were used to assess implementation quality immediately following implementation, and the 104 forms collected between December 2019 and February 2020 were used to assess sustainment. These forms were reviewed by an obstetrician on the Kybele training team to evaluate whether the assigned risk category and the care plan aligned with the patient’s clinical data collected at triage.

#### Sustainment data

Sustainment was measured in two ways. First, data on the outcomes and implementation quality was collected 12 months after implementation, as mentioned. Second, a questionnaire based on *normalization process theory (NPT)* [15–17] was administered to determine the extent to which triage practices became embedded in the midwifes’ routine clinical practice. A version of this questionnaire, called NoMad [18, 19], has been used in other global health settings and was adapted for obstetric triage. Approximately a year after implementation was completed, the questionnaire was administered in online and paper form to all individuals in the facility who acknowledged being connected to obstetric triage. This included midwifes, senior midwifes, nurses/enrolled nurses, senior nurses, shifts in-charge, and unit/ward heads. The questionnaire had three parts. The first part collected demographic and experience data. The second part queried the extent to which each step of the triage process (assessment, banding, triage form completion, care planning) was being routinely followed. The third part covered perceptions of the implementation organized by the four constructs of normalization: (1) *coherence*, which measures how the staff understand triage; (2) *cognitive participation*, or the extent to which participants are engaged and committed to triage implementation; (3) *collective action*, or the collective work taken on by participants to make triage work; and (4) *reflexive monitoring*, or how participants reflect on what triage has contributed to the organization [20]. Details of the questionnaire are presented in Supplemental File 2. A summary of the evaluation data collected is presented in Table 2.

**Table 2:** Outcome, implementation quality, and sustainment indicator summary

Data	Implementation stage	Dates	Source	Number of observations
**Arrival to assessment time**	Baseline	August 22–September 9, 2018	Observational admission-to-assessment time	66
	Implementation	November 27, 2018–January 12, 2019	Triage assessment forms	102^a^
	Post implementation	December 2, 2019–February 29, 2020	Triage assessment forms	104
**Banding accuracy**	Baseline	-	-	-
	Implementation	November 27, 2018–January 12, 2019	Triage assessment forms	103
	Post implementation	December 2, 2019–February 29, 2020	Triage assessment forms	104
**Care plan accuracy**	Baseline	-	-	-
	Implementation	November 27, 2018–January 12, 2019	Triage assessment forms	103
	Post implementation	December 2, 2019–February 29, 2020	Triage assessment forms	104
**Normalization**	Baseline	-	-	-
	Implementation	-	-	-
	Post implementation	March 2020	Normalization process theory assessment	60

^a^One time point was incomplete in the sample

### Data analysis

The primary outcome, the time from arrival to assessment, was analyzed in two ways: (a) by the median and interquartile range and (b) by the proportion of mothers who were assessed within 10 min of arrival (10 min is the recommended benchmark from time of arrival to time of assessment) [21]. Implementation quality was measured by proportions of mothers with accurate banding and care planning. To compare outcomes between the end of active implementation support and the sustainment period 1 year later, the two-sample Kolmogorov–Smirnov test for equality of distributions was employed. This test was utilized because it permitted comparison of the overall distributions, not just medians, providing a more comprehensive comparison of changes in the distribution of outcomes.

With the sustainability data from the NoMad assessment, demographic data were analyzed to understand the spread of respondents’ experience. In line with recommendations from NoMad developers, researchers calculated the percent of respondents who agreed or strongly agreed with each survey item [22]; generally speaking, the higher the score an item receives, the more likely the intervention is to be normalized within the given context. Two indicators included in the NPT assessment were converted mathematically to give all indicators the same directionality. Additionally, mean composite scores for each NPT construct (coherence, collective action, cognitive participation, and reflexive monitoring) were calculated to understand how many respondents agreed or strongly agreed across all indicators within each construct.

### Ethics approval and consent to participate

The study was declared exempt by the University of North Carolina’s Institutional Review Board.

## Results

### Outcome

Patient waiting time significantly decreased across the phases of the study. At baseline, the median arrival-to-assessment waiting time was 70.5 min (IQR: 30.0–443.0 min). Waiting time decreased to 6.0 min (IQR: 3.0–15.0 min) following active implementation and to 5.0 min (IQR: 2.0–10.0 min) during the sustainment period. The proportion of patients seen within 10 min of arrival are shown in Table 3. The difference in wait time distribution between active implementation and sustainment was statistically significant (combined K-S: *D* = 0.222, *p* = 0.018).

**Table 3:** Arrival-to-assessment time, accuracy of banding, and adequacy of care plan

	**Baseline**		**Implementation**		**Sustainment**		
	**% (*n*)**	**Total**	**% (*n*)**	**Total**	**% (*n*)**	**Total**	***p***
**Assessed within 10 min of arrival**	18.2% (12)	66	64.2% (66)	102	84.6% (88)	104	0.018
**Accurate band color assigned**	No data	No data	75.7% (78)	103	77.9% (81)	104	0.715
**Appropriate care plan developed**	No data	No data	66.0% (67)	103	78.9% (82)	104	0.003

### Implementation quality

Table 3 also shows the results of the implementation quality indicators of banding and care plan accuracy. There were no a priori benchmarks for these indicators, but the correctness of banding remained consistent, and care plan accuracy improved significantly a year after implementation.

Sixty-four staff members completed the NoMad questionnaire; of those, four were not familiar with OTIP, so 60 assessments were analyzed. There is substantial diversity in experience, role, and area of work responsibility among the respondents (Table 4), providing a good representation of the overall workforce.

**Table 4:** Profile of NoMad questionnaire respondents

Demographic characteristic	*N*	%
**Years of experience at facility**	**56**^**a**^	
< 1 year	11	20%
1–2 years	6	11%
3–5 years	11	20%
6–10 years	11	20%
11–15 years	8	14%
> 15 years	9	16%
^a^*Four respondents did not answer this question*		
**Current role**	**57**^**a**^	
Senior midwife	16	28%
Midwife	15	26%
Nurse/enrolled nurse	7	12%
Unit/ward head	7	12%
Physician	6	11%
Senior nurse	2	4%
Shift in charge	2	4%
Consultant, specialist	1	2%
Other	1	2%
^a^*Three respondents did not answer this question*		
**Current ward**	**58**^**a**^	
Labor and delivery	17	29%
Postnatal	13	22%
Theatre	10	17%
Antenatal	3	5%
Not assigned	2	3%
Multiple wards	2	3%
Other	11	19%

^a^Two respondents did not answer this question

Table 5 represents the respondents’ perceptions of the extent to which key steps of the triage process remained integrated into standard practice 1 year following program installation. There is some deterioration in compliance across the process steps, but overall, the staff feel that the triage program has been sustained.

**Table 5:** Utilization of obstetric triage system components

	**Always**	**Often**	**Sometimes**	**Rarely**	**Never**	**Do not know/missing**
Assessing patients as soon as possible after arrival	88.3%	6.7%	1.7%	0.0%	0.0%	3.3%
Banding patients according to risk	71.7%	13.3%	3.3%	0.0%	6.7%	5.0%
Completing the triage assessment form	65.0%	15.0%	5.0%	1.7%	5.0%	8.3%
Creating a risk-appropriate care plan	63.3%	18.3%	6.7%	1.7%	5.0%	5.0%

**Fig. 1:**
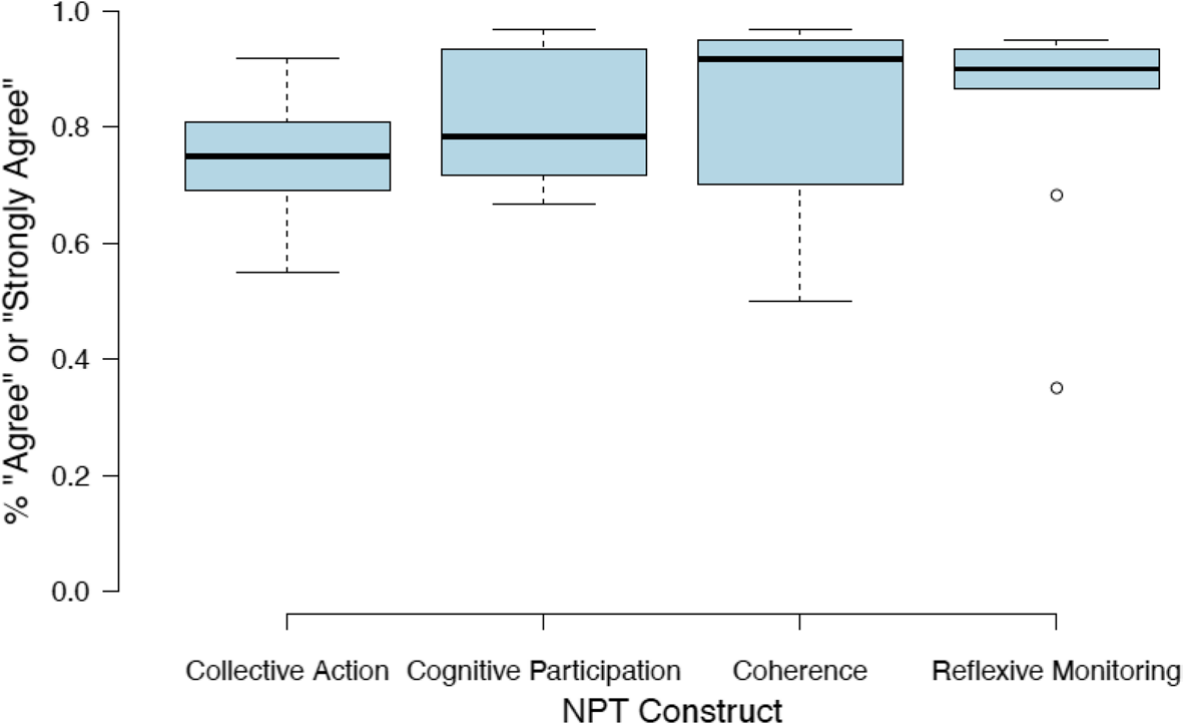
Percent of respondents who “agree” or “strongly agree,” by NPT construct

Figure 1 summarizes results of the last part of the NoMad questionnaire. The boxplots summarize the percentage of respondents who answered “strongly agree” or “agree” to each item in the survey organized by the four constructs of the questionnaire. A higher percentage reflects greater “normalization” of triage in the institution. Table 6 shows the definition of each construct, the number of items, and an extraction of the item level data indicating the top two and bottom two scoring items for each construct [20]. The complete table with scores for all items is included in the Supplementary files.

**Table 6:** Top two and bottom two scoring items for each NPT construct

NPT construct	Construct definition	Item	% agree and strongly agree
**Collective action (12 items)**	The work that is needed as a group to make obstetric triage work	I have easily integrated obstetric triage into my everyday work	91.7%
		Staff believe that they have the ability to do obstetric triage	86.7%
		There are regular reviews, or refresher trainings, of the obstetric triage process	65.0%
		There is an established process for replenishing triage bands	55.0%
**Cognitive participation (7 items)**	Level of commitment and engagement by implementers	Staff believe that participating in obstetric triage is part of their role as health providers	96.7%
		Obstetric triage will continue to be part of my everyday work in caring for mothers	93.3%
		Staff believe that there are opportunities to discuss implementation challenges and best practices with peers and champions	66.7%
		Staff believe that there are regular reviews of obstetric triage practices by coaches	66.7%
**Coherence (4 items)**	Meaning and purpose of obstetric triage to staff	I believe that obstetric triage has resulted in improved outcomes for mothers in my hospital	96.7%
		Staff see the value of obstetric triage in their work	93.3%
		Staff have a shared understanding of the purpose of obstetric triage	90.0%
		Staff believe that obstetric triage differs from how we prioritized high risk mothers in the past	50.0%
**Reflexive monitoring (10 items)**	Participants’ appraisal of obstetric triage	Obstetric triage will continue to be a normal part of work in my hospital in the future	95.0%
		Staff believe that obstetric triage is a normal part of our work	95.0%
		Staff are aware of reports or data about the effectiveness of obstetric triage	68.3%
		There is a process for selecting and training new champions	35.0%

Figure 1 indicates that overall, hospital staff perceive obstetric triage to be well integrated into the facility. Even in the lowest aggregate scoring construct, *collective action*, 75% or more of the respondents agree with 6 out of the 12 items, and as seen in Table 6, more than half agree with the lowest scoring item in this construct. The medians are even higher in the other constructs. For example, 90% or more of the respondents agree with 3 out of the 4 items for the *coherence* construct (Table 6).

### Process monitoring

**Fig. 2:**
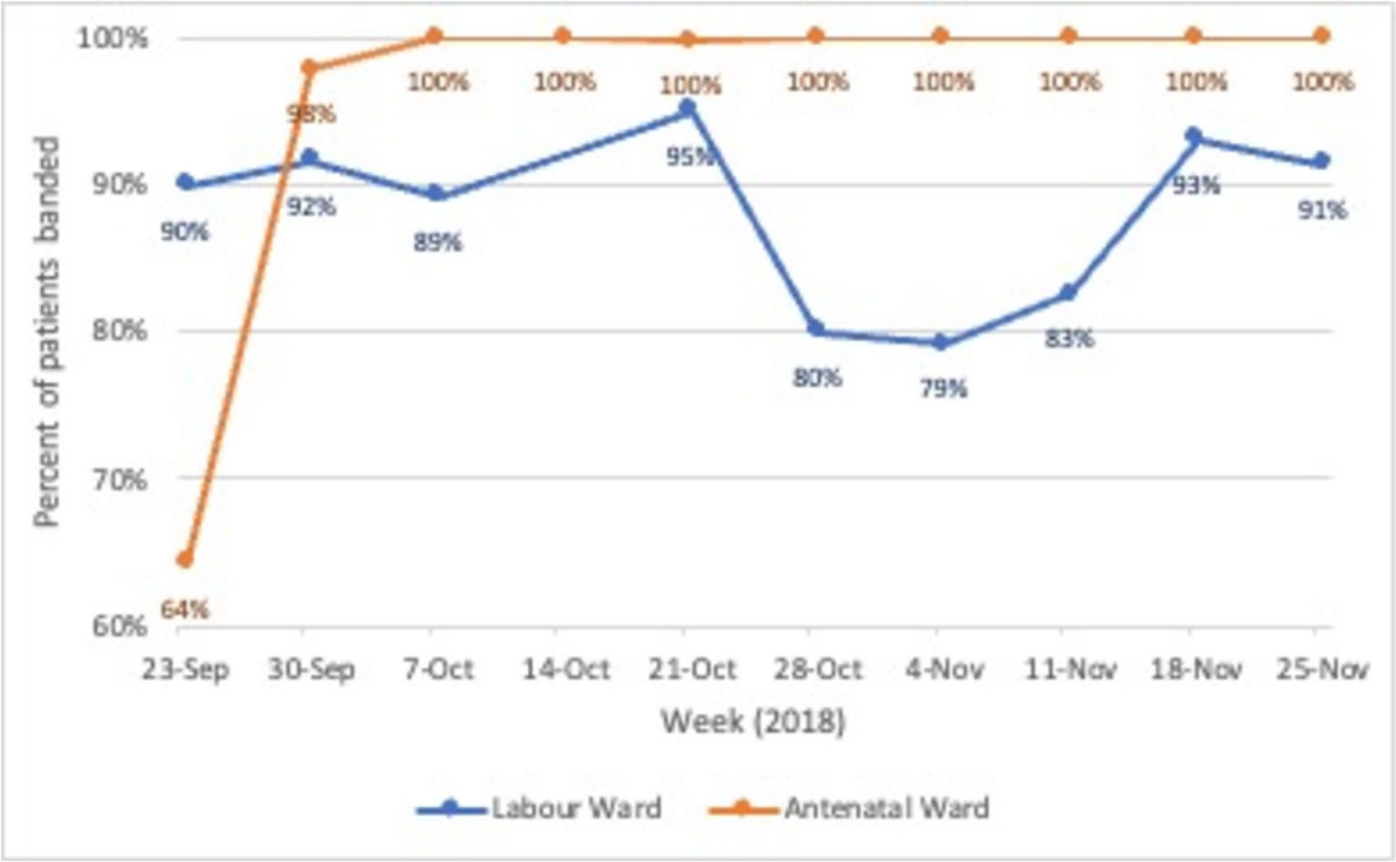
Average percent of patients banded by week

**Fig. 3:**
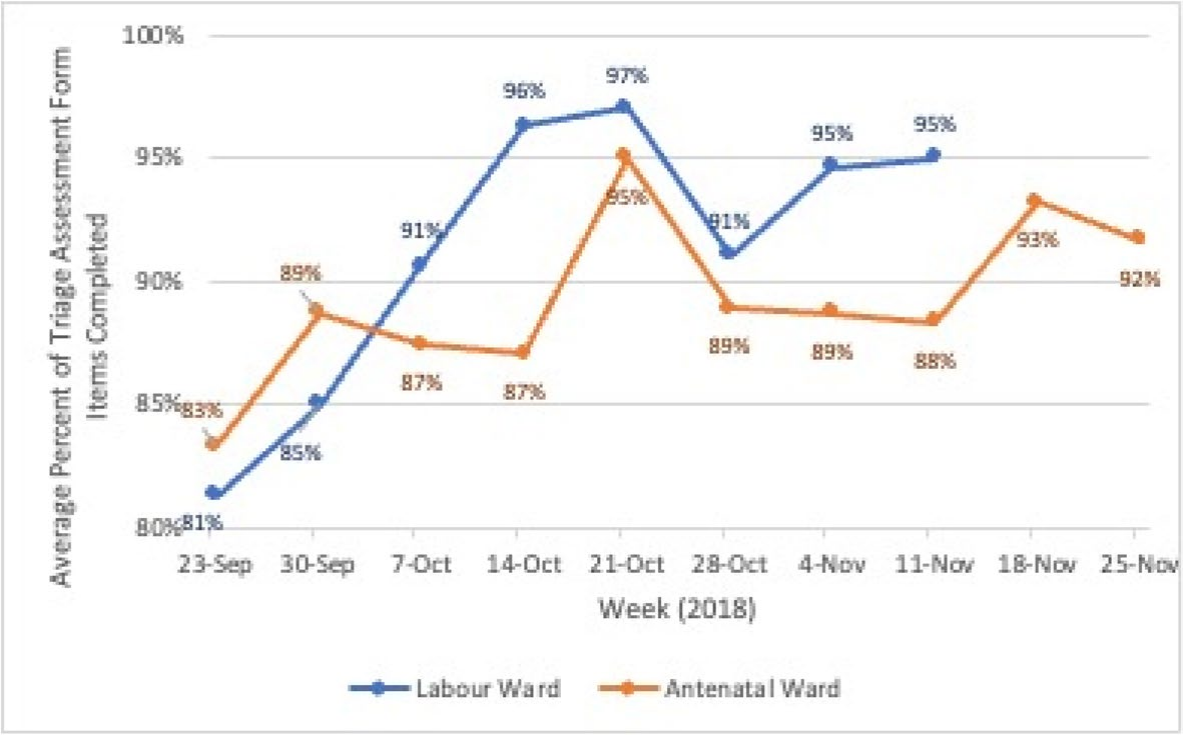
Average percent of triage assessment form items completed, per week

The process monitoring results are shown in Figs. 2 and 3 for the implementation compliance indicators shown in Table 1. As indicated, these data were tracked weekly to evaluate the progress of implementation and to take real time action as needed to improve implementation. These results show how routine monitoring provided the champions with a systematic approach to manage implementation and to motivate and challenge the staff to improve.

## Discussion

### Contribution to the field

The implementation of obstetric triage described in this paper has attempted to address several areas where there is on-going discussion and debate in implementation science. The first is the need for greater use of systematic implementation strategies in practice settings that can lead to the development of theories of implementation. Birken and others have acknowledged challenges that “*underuse, superficial use, and misuse of theories*” pose for implementation science [23]. Kislov proposes that implementation researchers adopt a “theorizing” approach where empirical data at a project or program level is continually used to test and enhance program specific theories with the goal of creating mid-range theories of implementation that are broadly applicable across multiple contexts [24]. We have followed such an approach. This implementation built on our initial experience with obstetric triage implementation at GARH, where an informal approach to implementation support (monitoring and improvement) developed as part of a QI process showed promise in facilitating the achievement of outcomes [7]. In this facility, the implementation support activities were systematized into a more formal protocol, situated within the existing literature on implementation strategies [11], and implementation outcomes were measured. This assessment established that the strategy to build capacity for monitoring and improvement was acceptable and feasible for programs of this type and resulted in the desired outcomes. The empirical data from this evaluation enabled the creation of a more advanced implementation theory for a future program (conducted between 2019 and 2022) to scale obstetric triage to six hospitals in Ghana. This theory, published elsewhere [25], is based on the Evidence-Based System for Implementation Support (EBSIS) [9] that specifies the need for training to be reinforced by technical assistance and quality improvement and on the leadership literature authored by Aarons et al. identifying the critical role of leaders in supporting implementation [26, 27]. The expectation is that the evaluation of this theory (currently underway) will enable the creation of a mid-level theory to implement similar QI driven programs to improve health outcomes in low resource settings.

Our study also illustrates one approach to integrate the fields of quality improvement and implementation science. Over the years, there has been tension between the two fields despite their common goal of improving outcomes across healthcare and population settings. Recently, there have been efforts to systematically compare the two fields and to advocate for their integration [28, 29]. Our study demonstrates how this can be done in practice. As mentioned, the original implementation at GARH was based on the Model for Improvement, which was used to design, test, and adapt the components of the clinical intervention (e.g., the colored bands, the triage assessment forms, etc.). For this implementation, the model was adapted to develop an implementation hypothesis, implementation outcome indicators for fidelity and sustainability (measured during the implementation process), and acceptability, feasibility, and adoption (measured using the NPT survey). Specifically, our approach demonstrates how the core concepts of implementation science (implementation outcomes, determinants, and implementation strategies) are complementary to those of QI (improvement aims, change drivers and change solutions) and that both set of concepts are necessary to create, instantiate, and sustain context-appropriate interventions in clinical settings. Our insights from triage implementation have led to the creation of an integrated model called the Model for Improvement and Implementation that provides guidance on how to jointly optimize adaptations and implementation strategies to create implementation informed interventions that are more likely to be adopted and sustained [30].

Third, our study demonstrates the application of an implementation strategy to an intervention that is a *system* consisting of various sequential steps, each of which need to be completed with quality in order for the intervention to be successful. The language of systems change is central to quality improvement [31] but is not common in implementation research where interventions are typically described as having single or multiple components but are not specifically linked to care delivery process steps [32]. By explicitly situating our implementation strategy within the pathway of providing care to pregnant mothers, our approach allows us to systematically ensure that all critical activities needed to achieve outcomes are implemented with fidelity. In this project, the same implementation strategy was used for all steps of the triage process, but it is conceivable that different steps might lend themselves to different strategies.

Finally, our study measures sustainment, which is largely missing in implementation research studies, yet has been identified as a critical priority for the field [33, 34]. A recent literature review examined 791 studies and their use of implementation research principles in LMICs; findings indicate that only 3% of studies addressed sustainability [35]. It is estimated that only half of the health-related, evidence-based interventions that are implemented are sustained over time [36–38]. Yet, there is no approach to measuring sustainment that is a common standard. Our results indicate that the use of a systematic implementation approach that is acceptable and feasible can result in sustainment for at least 12 months. The NPT questionnaire provides some key learning about factors that affect sustainment.

### Determinants of sustainment

One learning relates to the effectiveness of using quality monitoring by front line staff selected and trained to be champions, as an implementation strategy. The last column of Table 3 shows the sustainment of the triage program on the outcome and implementation quality indicators. The first two columns of Table 5 show the percentage of staff who report following the key process steps resulting in these outcomes “always” or “often.” There is an encouraging concordance between these two percentages (e.g., 85% or patients are assessed within 10 min of arrival, and 95% of the staff report a commitment to quick assessment). This strengthens our level of confidence that training and supporting staff to use data to identify implementation challenges and to give them the tools and authority to solve problems on their own facilitates the adoption and sustainment of an intervention.

The NPT item-level analysis also provides critical insights into factors that may have contributed to the successful sustainment of OTIP. There are two overarching themes of NPT constructs that staff strongly agreed or agreed with: (1) obstetric triage was easily integrated into their everyday work and seen as a normal part of their role and (2) obstetric triage was viewed as a worthwhile program and something that positively impacted staff and patients. These findings resonate with existing research, indicating that a key determinant of implementation success is whether intervention users—in this case, facility staff—perceive the new intervention as relevant or important [39].

In general, the staff are confident about items that reflect their own ability and motivation but are less secure in the support they get from the institution. Existing literature indicates that both elements—staff motivation and confidence, as well as systematic or institutional support—are critical for achieving sustainability [33, 40]. Across the constructs, 92% of the respondents feel that they have integrated triage into their work, 97% feel that it is part of their role, 97% feel that it has improved outcomes for mothers, and 95% feel that it will continue to be part of their work in the future. In contrast, there is a sense that any failures in sustainment come from system issues such as lack of processes for recruitment, training, review, and supplies. The NPT results identified the following barriers to sustainment: (1) perceived gaps in facility providing appropriate space, equipment, and supplies for triage (i.e., replenishing bands, printing forms) and (2) lack of opportunity for ongoing learning opportunities related to triage, including refresher trainings, review of monitoring data, opportunities to discuss implementation challenges, and ongoing coaching and mentoring. It is not inconceivable that, in the longer run, failure on the part of the health system leadership to attend to these barriers could jeopardize the sustainability of the program. Bands and forms are the critical supplies that make triage possible. Frequent staff turnover in these facilities and the lack of established refresher training could affect the knowledge, skills, and confidence of new staff. Our opinion is that strengthening the system is important if the sustainment that has been achieved is to be maintained.

### Limitations

There are several limitations that may affect both our conclusions about program implementation and the overall impact of triage. First, this is was single site implementation that enabled the Kybele implementation team to be closely involved in the implementation support and to build personal relationships with the triage champions and with the head of obstetrics. This provided an enabling environment for the champions to adopt the implementation strategy which has significant data collection and analysis requirements. Second, the triage champions at this facility were already senior leaders in their wards and had the respect of the staff and the authority to bring about change. It is difficult to disentangle the effect of the implementation strategy from the effect of leadership strength, especially because many of the solutions to implementation challenges were not changes to the system to improve implementation, but primarily involved ongoing encouragement, reinforcement, and coaching of staff.

Also, the success of triage and the achievement of the primary outcome of the program (how quickly mothers are seen) may not result in any change to maternal outcomes that matter, such as mortality, or appropriate and timely care. The triage program ends with the creation of a care plan, but the plan needs to be acted upon through labor and delivery, and any changes to the mothers’ risk level needs to be noted and re-banding has to take place. While the triage program is midwife-led, downstream conduct of the care plan needs to involve both doctors and midwives, and there was not much involvement of doctors in the design and implementation of the triage program. We have anecdotal data that indicates that downstream care was not always compliant with the risk level, but the extent and impact of this is unknown. Future implementation efforts need to explicitly involve other stakeholders including doctors and facility leaders so that the critical value of triaging high-risk mothers extends to their entire process of care.

Finally, the NPT data was self-reported, and it is possible that the close relationship between the implementers (who also evaluated the program implementation) may have resulted in desirability bias that affected the results. Follow up interviews with staff asking for explanations for high and low scoring items, and those that had the greatest variation among the respondents would have provided more insights about the scores, but COVID-19 travel restrictions made that infeasible. This is an important area of exploration for future administrations of the NPT survey. In addition, while the implementation was sustained a year after formal implementation support ended, the importance of the systems factors mentioned in the survey (a process for recruiting new champions, training for new staff, ongoing coaching and support etc.) for ongoing sustainment cannot be minimized. This program did not explicitly build in these systems strengthening activities that could influence its sustainability over time.

## Conclusion

The need for more systematic use of theory in implementation research to increase rigor in the field is gaining prominence. There is an acknowledgement that the inherent theoretical base of the field is weak, attributable in part to its newness [23, 41, 42]. NPT, one of the few recognized theories in implementation science [4], demonstrates its use in practice. We did not use a formal theory driven approach such as implementation mapping for the generation of the implementation strategy [43], but we use what Kislov calls a “theoretically informative” approach which develops theories through iterative testing and refinement in a real life setting [24]. The triage implementation using a capacity building implementation strategy led by midwives has shown promise in achieving and sustaining outcomes, and this has served as the basis for the next iteration of the theory that we have developed for scaling up the triage program in several other facilities [25]. This study has made a contribution both to addressing the important issue of providing timely care to high-risk women and to advancing methods in implementation science.

### Supplementary Information

**Additional file 1: Supplemental File 1.** Triage champion selection criteria. **Supplemental File 2.** Percent of respondents who agree or strongly agree with each NPT variable

## Data Availability

The datasets used and/or analyzed during the current study are available from the corresponding author on reasonable request.
