# Supplementary material for: Implementation matters: assessing the effectiveness and sustainment of an obstetric triage program at a high-volume facility in Ghana
Source: Implement Sci Commun. 2023 Nov 15;4:138. doi: 10.1186/s43058-023-00527-y (PMC10647175; doi:10.1186/s43058-023-00527-y)
Supplement: Supplementary file 1 — Additional file 1: Supplemental File 1. Triage champion selection criteria. Supplemental File 2. Percent of respondents who agree or strongly agree with each NPT variable [file 43058_2023_527_MOESM1_ESM.docx]

**SUPPLEMENTAL MATERIALS**

**Supplemental File 1.** Triage champion selection criteria

The role of the Triage Champion will be to introduce, establish, manage, and sustain an obstetric triage system as a functioning part of the hospital maternity services. They will provide training in obstetric triage for midwives and other involved parties, monitor and evaluate the triage quality and ensure successful implementation and integration into the maternity unit. The triage clinical champion will report to the head of OBGYN, the director of nursing services, and the triage implementation team.

**Responsibilities**

- Set up and equip an obstetric triage area.
- Liaise with other professionals about the obstetric triage service to explain how it will be conducted within the everyday workflow.
- Provide training for midwives and other parties in obstetric triage and communicate its importance in delivering safe obstetric care.
- Introduce and supervise use of the obstetric triage tool kit, colour-coded wristbands, triage risk acuity chart, and the triage assessment form.
- Conduct and oversee quality triage on a day-to-day basis. Ensure that equipment is functioning and that there are adequate supplies and medications, wristbands and triage assessment forms.
- Monitor and evaluate the service by collecting data on the number of patients with wristbands, completion of triage assessment form, and correct wristband colour. Monitoring patient waiting times and formulate plans of care. This will be done on a weekly basis and communicated to the implementation team.
- Assess patient satisfaction with service.

**Qualifications**

- Registered midwife
- Demonstrates the ability to be a good leader
- Works well in a team
- Demonstrates flexibility and the ability to handle stressful situations
- Demonstrates good communication skills at all levels with staff, patients, families, senior staff, and management
- Shows understanding of compassionate care
- Is able to take the initiative in difficult situations

**Experience**

- Four years or more of midwifery experience, having worked in antenatal, labour and delivery, and postnatal areas
- Understands the pathway women take in the course of pregnancy and delivery as well as the concept of the high and low-risk patient and how this may change during pregnancy and labour
- Competent in initial assessment and management of obstetric emergencies
- Knows when and how to refer a patient for a higher level of care
- Is competent in computer use, collecting and recording of data

**Supplemental File 2.** Percent of respondents who agree or strongly agree with each NPT variable

| **NPT Construct** | | **Question** | **% Agree & Strongly Agree** |
| --- | --- | --- | --- |
| **Collective Action (12 items)** | I have easily integrated obstetric triage into my everyday work | | 91.7% |
|  | Staff believe that they have the ability to do obstetric triage | | 86.7% |
|  | Staff believe that obstetric triage has negatively affected work in our facility* | | 81.7% |
|  | Staff believe that new staff are being trained and supported in how to do obstetric triage | | 80.0% |
|  | Staff believe that the leadership is committed to obstetric triage | | 78.3% |
|  | A process exists for ongoing monitoring for banding compliance and accuracy | | 75.0% |
|  | Staff believe that the facility provides the necessary space, equipment and supplies to support obstetric triage | | 73.3% |
|  | There is an established process for printing triage forms | | 70.0% |
|  | The facility provides the necessary space, equipment and supplies to support obstetric triage | | 68.3% |
|  | There are regular reviews, or refresher trainings, of the obstetric triage process | | 65.0% |
|  | There is an established process for replenishing triage bands | | 55.0% |
| **Cognitive Participation (7 items)** | Staff believe that participating in obstetric triage is part of their role as health providers | | 96.7% |
|  | Obstetric triage will continue to be part of my everyday work in caring for mothers | | 93.3% |
|  | Staff believe that they contribute to ensuring obstetric triage is successful | | 93.3% |
|  | Staff believe that champions ensure people are participating in obstetric triage | | 78.3% |
|  | Staff believe that they receive mentoring from triage champions when needed | | 76.7% |
|  | Staff believe that there are opportunities to discuss implementation challenges and best practices with peers and champions | | 66.7% |
|  | Staff believe that there are regular reviews of obstetric triage practices by coaches | | 66.7% |
| **Coherence (4 items)** | I believe that obstetric triage has resulted in improved outcomes for mothers in my hospital | | 96.7% |
|  | Staff see the value of obstetric triage in their work | | 93.3% |
|  | Staff have a shared understanding of the purpose of obstetric triage | | 90.0% |
|  | Staff believe that obstetric triage differs from how we prioritized high risk mothers in the past | | 50.0% |
| **Reflexive Monitoring (10 items)** | Obstetric triage will continue to be a normal part of work in my hospital in the future | | 95.0% |
|  | Staff believe that obstetric triage is a normal part of our work | | 95.0% |
|  | I believe obstetric triage is a worthwhile program | | 93.3% |
|  | Obstetric triage still feels very new in my hospital* | | 90.0% |
|  | Obstetric triage is now a normal part of work in my hospital | | 90.0% |
|  | Obstetric triage has positively affected my everyday work in caring for mothers | | 90.0% |
|  | Staff believe that obstetric triage is a worthwhile program for our facility | | 88.3% |
|  | Staff believe that obstetric triage has improved the relationships between doctors and midwives | | 86.7% |
|  | Staff are aware of reports or data about the effectiveness of obstetric triage | | 68.3% |
|  | There is a process for selecting and training new champions | | 35.0% |

*Indicators were mathematically converted to give all indicators the same directionality.
